# Functional deletion of α7 nicotinic acetylcholine receptor impairs Ca^2+^-dependent glutamatergic synaptic transmission by affecting both presynaptic and postsynaptic protein expression and function

**DOI:** 10.3389/fphys.2025.1662171

**Published:** 2025-08-08

**Authors:** Beatrice Cannata, Laura Sposito, Martina Albini, Giuseppe Aceto, Giulia Puliatti, Giacomo Lazzarino, Cristian Ripoli, Maria Rosaria Tropea, Daniela Puzzo, Roberto Piacentini, Claudio Grassi

**Affiliations:** ^1^ Department of Neuroscience, Università Cattolica del Sacro Cuore, Rome, Italy; ^2^ Fondazione Policlinico Universitario A. Gemelli IRCCS, Rome, Italy; ^3^ Departmental Faculty of Medicine, UniCamillus - Saint Camillus International University of Health Sciences, Rome, Italy; ^4^ IRCCS San Camillo Hospital, Venice, Italy; ^5^ Department of Biomedical and Biotechnological Sciences, University of Catania, Catania, Italy; ^6^ Oasi Research Institute-IRCCS, Troina, Italy

**Keywords:** α7 nAChRs, NMDA, AMPA, hippocampus, glutamate, acethylcholine

## Abstract

Alpha7 nicotinic acetylcholine receptors (α7-nAChRs) are ionotropic, Ca^2+^-permeable receptors highly expressed in brain regions involved in memory formation, such as the hippocampus. Their activation induces cation influx and neuronal depolarization, which in turn promotes glutamate release—highlighting a crucial interplay between cholinergic and glutamatergic signaling in the healthy brain. Interestingly, the genetic deletion of α7-nAChRs in mice (α7-KO mice) leads to an Alzheimer’s disease (AD)-like phenotype characterized by aberrant amyloid-β accumulation, tau phosphorylation, and neuroinflammation in aged (>12 months) mice. However, glutamatergic transmission in these mice prior to the onset of the AD-like phenotype has been poorly investigated. We thus studied molecular and functional properties of glutamatergic transmission in 4–6-months-old α7-KO mice, representing a prodromal phase of the AD-like neuropathology. We found that hippocampal CA1 neurons in brain slices from α7-KO mice showed a reduced frequency of the spontaneous excitatory post-synaptic currents (sEPSCs) compared to those of wild-type (WT) mice. On the contrary, the amplitude of sEPSCs was not affected, although in α7-KO neurons these currents displayed a longer rise time than in wild-type. CA1 neurons from α7-KO mice also exhibited a significantly smaller evoked NMDA currents than WT neurons, whereas AMPA currents were unaffected. From a molecular point of view, hippocampal neurons of α7-KO mice exhibited an increased expression of the pre-synaptic protein Synapsin-1 and of the NMDA subunits GluN2A and GluN2B, but no modifications in the expression of AMPA receptor subunits (GluA1 and GluA2) were found. Importantly, selective re-expression of the α7-nAChRs in neurons of α7-KO mice restored the evoked NMDA current amplitude and the rise time of sEPSCs, but it did not rescue the frequency of sEPSCs, thus suggesting that post-synaptic integrity depends on neuronal α7-nAChRs.

## Introduction

Alzheimer’s disease (AD) is the most common neurodegenerative disorder and the leading cause of dementia, accounting for at least two-thirds of cases in individuals aged 65 and older worldwide (Kumar et al., 2025). Characterized by an insidious onset and progressive decline in behavioral and cognitive functions, AD significantly impairs daily functioning. Several studies have identified amyloid-β (Aβ) and hyperphosphorylated tau (pTau) proteins as key contributors to AD pathology due to their accumulation in insoluble aggregates and soluble oligomeric forms, with the latter considered primarily responsible for early synaptic failure (Selkoe, 2002; Spires-Jones and Hyman, 2014), especially at hippocampal level (Henstridge et al., 2019).

Although glutamatergic transmission is primarily affected in both early and late stages of AD (Zott and Konnerth, 2023), a loss of function of nicotinic acetylcholine receptors (nAChRs) — particularly the homopentameric α7 isoform, which is the most abundant subtype expressed in the mammalian hippocampus—has also been hypothesized to contribute to AD pathogenesis (Ferreira-Vieira T et al., 2016; Parri et al., 2011; Wu et al., 2010; Yakel, 2012; Rao et al., 2022). Indeed, an important cross-talk between cholinergic and glutamatergic transmission has been reported (Francis, 2003). The ionotropic α7-nAChR promotes Ca^2+^ influx, regulating activation of several kinases and gene transcription (Abraham et al., 2025), and leading to membrane depolarization and enhanced glutamatergic excitatory signaling by triggering glutamate release from presynaptic terminals. This process contributes to synaptic plasticity, such as long-term potentiation (LTP) at CA3-CA1 synapse, and memory formation (Cheng and Yakel, 2015). Notably, mice lacking α7-nAChRs (α7-KO) develop an age-dependent AD-like phenotype, with accumulation of AD hallmarks, neuroinflammation, and hippocampal synaptic plasticity and memory deficits starting from about 12 months of age (Tropea et al., 2021). However, glutamatergic transmission in these mice prior to the onset of the AD-like phenotype remains poorly understood. Based on these premises we aimed to investigate molecular and functional basis of glutamatergic transmission in α7-KO mice aged 4–6 months, corresponding to a prodromal phase of the AD-like neuropathology. In addition, we employed AAV-mediated gene re-expression to selectively restore neuronal α7-nAChR expression in α7-KO mice, in order to dissect its causal role in the glutamatergic deficits exhibited by this genetic model.

## Materials and methods

### Animal models and ethics approval

We used wild type (WT, C57BL/6; RRID: IMSR_JAX:000664) and α7-KO mice (B6.129S7-Chrna7^tm1Bay^/J; RRID: IMSR_JAX:003232) purchased from The Jackson Laboratory. Colonies were established in the animal house of Università Cattolica del Sacro Cuore. Housing conditions were controlled maintaining stable hygrometric and thermic conditions (50%; 21°C ± 1°C) on 12 h light/dark cycle with *ad libitum* access to food and water. Mice were used at 4–6 months of age, and were sex balanced. All animal procedures were approved by the Ethics Committee of Università Cattolica and Italian Ministry of Health (authorization n. 944/2021-PR) and were fully compliant with Italian Ministry of Health guidelines (Legislative Decree No. 116/1992) and European Union (Directive No. 2010/63/EU) legislations on animal research. All experiments were conducted to minimize animal suffering.

### Primary cultures of hippocampal neurons

Primary cultures of neurons were prepared from hippocampi of wild-type (WT) E18 C57BL/6 and B6.129S7-CHRNA7 (α7-KO) mice, as previously described (Tropea et al., 2021; Piacentini et al., 2017; Li Puma et al., 2022). Briefly, brain tissues were gently dissected in Phosphate Buffered Saline (PBS) at 4°C and then incubated for 10 min at 37°C in PBS containing trypsin–ethylenediaminetetraacetic acid 0.025%/0.01% w/v (Trypsin-EDTA, Biochrom AG, Berlin, Germany). After trypsin inactivation through fetal bovine serum (FBS), tissues were centrifugated and resuspended in the dissociation medium, consisting of minimum essential medium (MEM, Biochrom) supplemented with 1% FBS, 2 mM glutamine, 25 mM glucose, and 1% penicillin–streptomycin-neomycin antibiotic mixture (PSN, Thermo Fisher Scientific, Waltham, MA). Tissues were mechanically dissociated with a fire-polished Pasteur pipette at room temperature (RT) and then centrifuged at 235 × g for 10 min at RT. Cells were resuspended in the previously described medium added with 5% horse serum and 5% FBS and plated on poly-L-lysine (0.1 mg/mL, Sigma, St. Louis, MO)-pre-coated 20-mm coverslips (10^5^ cells/well) for confocal Ca^2+^ imaging and on 35-mm six-well plates (10^6^ cells/well) for High-Performance Liquid Chromatography (HPLC) and Western blot (WB) analyses. After 24 h from seeding (1 day *in vitro*), the culture medium was replaced with a fresh medium consisting of 96.5% Neurobasal medium (Thermo), 2% B-27 (Thermo), 2 mM glutamine and 1% PSN; after another 72 h (4 days *in vitro*), this medium was replaced with a glutamine-free version of the same medium, and the cells were grown for 10 more days before carrying-out experiments. Cell cultures were incubated in a 5% CO_2_ humidified incubator at 37°C.

### Organotypic hippocampal brain slices preparation

Hippocampal organotypic slice cultures were prepared from WT P4-8 C57BL/6 mice using a McIllwain tissue chopper, employing at least four mice for each preparation. Slices (300 µm) were placed on semi-porous membranes (Merck Millipore, No. PCIMORG50, Burlington, MA, United States) fed by tissue medium made of MEM (Thermo) supplemented with 30 µM HEPES, 5.8 mM NaHCO_3_, 26.6 mM D-glucose, 2.5% ascorbic acid, 0.5 mg/mL insulin, 20% horse serum (Thermo), 2 mM MgSO_4_, 1 mM CaCl_2_. Slices were incubated at 36°C in 5% CO_2_.

### Plasmid design and viral vector assembly for CHRNA7 overexpression or silencing

In order to overexpress CHRNA7 we used the purchased plasmid Addgene #62276. To generate plasmids capable of overexpressing CHRNA7 selectively in neurons, the CHRNA7 coding sequence from plasmid Addgene #62276 was amplified by PCR and inserted into two in-house pAAV2 viral backbones, containing the hSyn promoter as well as mRuby2 gene reporter, using Gibson Assembly (Gibson Assembly Master Mix, New England Biolabs, Ipswich, MA, United States). All restriction enzymes were purchased from New England Biolabs. In this way, we generated pAAV-hSyn-CHRNA7-mRuby2. Sequence verification was performed by Sanger sequencing. Data were analyzed using SeqScape Software (Applied Biosystems, Foster City, CA, United States), supported by SnapGene (GSL Biotech, Boston, MA, United States).

For the silencing of endogenous CHRNA7, we inserted a home-made CHRNA7-targeting shRNA sequence under the hSyn promoter into pAAV2 viral backbone using Gibson Assembly. The final constructs, carrying mRuby2 as a reporter, was named pscAAV [shRNA]-hSyn>{CHRNA7_shRNA}-hSyn > mRuby2, and allows the silencing selectively in neurons.

Finally, both the overexpression and silencing plasmids were turned into adeno-associated viral vector by InnovaVector s.r.l. (Pozzuoli, Italy), generating the following constructs:

• AAV2-PHP.eB-hSyn > CHRNA7-mRuby2
• AAV2-PHP.eB-hSyn > shRNA_CHRNA7-hSyn > mRuby2

### Viral infections

*Ex vivo*. Hippocampal organotypic slices from WT mice were infected 2 h after preparation with AAV2-PhP.eB-hSyn > shRNA_CHRNA7-hSyn > mRuby2 at a concentration of 2.8 × 10^10^ gc/slice. Slices were studied 1 week after the infection.

*In vivo*. Three-month old α7-KO mice were anesthetized (80–100 mg/kg ketamine + 5–10 mg/mL xylazine) and placed in the stereotaxic frame. AAV2-PhP.eB-hSyn > CHRNA7-mRuby2 vector was inoculated in the dorsal hippocampus at the following coordinates (2 µL/region): dorsal CA1: 2.1 AP, ±1.8 ML, – 1.5 DV; dorsal CA2: 2.1 AP, ±2.2 ML, – 1.9 DV; and dorsal CA3: 2.1 AP, ±2 ML, – 2.1 DV. At the end of the procedure, the surgical wound was sutured, and the animals were administered with saline solution (200 µL/mouse) via intramuscular injection. Three weeks after the injections, mice were sacrificed by cervical dislocation and their brain collected for WB analysis or electrophysiological experiments.

### Confocal Ca^2+^ imaging

To perform Ca^2+^ imaging, cultures of WT and α7-KO neurons were incubated for 30 min at 37°C with 2.5 μM Fluo-4-AM (Thermo), a Ca^2+^ sensitive fluorescent dye, in Tyrode’s solution. This solution consisted of 150 mM NaCl, 10 mM glucose, 10 mM HEPES, 4 mM KCl, 2 mM CaCl_2_ and 1 mM MgCl_2_, and its pH was adjusted to 7.4 with NaOH. Cells were then maintained in fresh Tyrode’s solution at RT for 20 min to allow dye de-esterification. Intracellular Ca^2+^ transients were elicited after cell depolarization obtained by exposing Fluo-4-AM-loaded cells to 50 mM KCl for 10 s. Fluo-4 was excited at 488 nm and its emission signal was collected between 500 and 550 nm with an inverted laser scanning confocal system Leica TCS-SP5 (Wetzlar, Germany). The amplitude of each Ca^2+^ signal was estimated in a semi-quantitative way by the following formula: ΔF/F = (F_t_–F_pre_)/(F_pre_–F_bgnd_), where F_t_ is the mean of fluorescence intensities measured in a region of interest (ROI) drawn around each cell body at a given time (t); F_pre_ is the basal fluorescence intensity in this ROI estimated as mean value of fluorescence during 20-s prior KCl exposure; F_bgnd_ is background fluorescence intensity measured in an area lacking dye-filled cells.

### High-performance liquid chromatography (HPLC)

For HPLC measurements, primary cultures of WT and α7-KO neurons were stimulated with 50 mM KCl for 30 s in Tyrode’s solution. After this treatment, supernatants were collected and treated as already done in (Piacentini et al., 2017; Li Puma et al., 2022; Puliatti et al., 2023). Tyrode’s solution was withdrawn from each well and deproteinized according to (Tavazzi et al., 2005). Briefly, supernatant samples were transferred to an Eppendorf tube equipped with a filtering membrane of 3 KDa cut-off (Nanosep® Centrifugal Devices, Pall Gelman Laboratory, Ann Arbor, MI, United States) and centrifuged at 10,500 × g for 15 min at 4 °C. The protein-free ultrafiltrate samples were analyzed by HPLC to determine extracellular glutamate concentrations released by neurons in culture media, by using an automated pre-column derivatization protocol, with a mixture of 25 mmol/L orthophthalaldehyde (OPA), 1% 3-methylpropionic acid (MPA) and 237.5 mmol/L sodium borate, pH 9.8, as described by Lazzarino et al. (2022). The HPLC apparatus consisted of a Surveyor HPLC System (Thermo Fisher Italia, Rodano, Milan, Italy) and a highly sensitive photodiode array detector, equipped with a 5 cm light path flow cell, set up between 200 and 400 nm wavelength for acquisition of chromatographic runs. Data were acquired and analyzed by ChromQuest® software package, version 5.0 provided by the HPLC manufacturer. Assignment and calculation of derivatized-glutamate levels in culture media were carried out at 338 nm wavelengths by comparing retention times and areas of peaks with those of chromatographic runs of freshly prepared ultra-pure standard containing known glutamate concentrations. In each cell culture (WT and α7-KO) the total amount of proteins was determined according to the Bradford method (Bradford, 1976). Glutamate levels in culture media were normalized for the total cell protein concentrations and expressed as nmol/mg of proteins.

### Protein isolation and western blot analysis

Cells cultures, hippocampal organotypic slices, and hippocampi were lysed in RIPA buffer, supplemented with 1 mM phenylmethylsulfonyl fluoride (PMSF), sodium fluoride (NaF), sodium orthovanadate (Na_3_VO_4_), and protease inhibitor (PI) mixture. Then, they were sonicated and centrifuged at 13,000 × g for 20 min at 4°C. The supernatants were collected, and their protein concentration was assessed by BCA Assay Kit (Thermo). For each sample, an equivalent amount of protein (30 µg) was loaded onto 8%–12% tris-glycine polyacrylamide gel for electrophoresis separation. Proteins were then electroblotted onto nitrocellulose membranes and blocked with 5% non-fat dry milk in tris-buffered saline containing 0.1% Tween-20 for 1 h at RT. Membranes were incubated overnight at 4°C with a combination of the following primary antibodies (all diluted 1:1,000): rabbit anti-nicotinic acetylcholine receptor alpha 7 (#ab216485, Abcam), rabbit anti-Synapsin-1 (#5297, Cell Signaling Technology, Danvers, MA, United States), mouse anti-Synaptophysin (#ab8049, Abcam), mouse anti-GluA1 (#MAB2263, Merck Millipore), mouse anti- GluA2 (#30–0300, Invitrogen), mouse anti-NMDAR2B (#610417, BD Biosciences, Franklin Lakes, NJ, United States), rabbit anti-NMDAR2A (#07–632, Merck Millipore), rabbit anti-Homer1 (#PA5-21487, Invitrogen), rabbit anti-SNAP25 (#5309, Cell Signaling). Mouse anti-GAPDH [1D4] (#A85382, Antibodies.com, Cambridge, United Kingdom) was used as loading control. Membranes were then incubated with appropriate secondary horseradish peroxidase-conjugated (HRP) antibodies diluted at 1:5,000 (anti-rabbit #7074, anti-mouse #7076; Cell Signaling) for 1 h at RT. Visualization was performed with WESTAR ECL (Cyanagen, Bologna, Italy), using UVItec Cambridge Alliance. Molecular weights for immunoblot analysis were determined through Precision Plus Protein™ Standards (BioRad, Hercules, CA). Densitometric analysis was carried out with UVItec software. Experiments were repeated at least three times. Original uncropped gel and/or Western blot analysis are available upon reasonable request.

### Immunohistochemistry

Immunofluorescence analyses were performed on coronal sections (40 μm thick) of perfused brains from α7-KO mice infected with the viral vector AAV2-PhP.eB-hSyn > CHRNA7-mRuby2 in the left hemisphere, whereas the contralateral one was used as an internal control. The slices were incubated for 15 min with DAPI (0.5 μg/mL) and the sections were mounted on glass slides and coverslipped with ProLong Gold antifade reagent. Confocal stacks of images were acquired with a confocal laser scanning system Nikon A1MP, detecting the reporter gene mRuby2 and DAPI signals.

### Electrophysiological recordings

Electrophysiological analyses were performed on *ex vivo* hippocampal slices as previously described by (Mainardi et al., 2017). For both evoked and spontaneous excitatory post-synaptic current (EPSC) recordings, neurons were voltage-clamped at −70 mV, and for evoked currents electrical stimuli were delivered to the Schaffer collaterals. To determine the stimulus intensity for evoked EPSCs, input–output curves were first generated to identify the maximal response amplitude. Subsequent recordings were conducted using stimulation intensity corresponding to 30% of the maximal response. To assess the AMPA/NMDA current ratio, stimuli of identical intensity were delivered at holding potentials of −70 mV and +40 mV, respectively, at a frequency of 0.05 Hz. For spontaneous EPSC (sEPSC) recordings, neurons were held at −70 mV throughout the acquisition. Recordings were obtained using a Digidata 1440A Series interface and pClamp 10 software (Molecular Devices). Signals were filtered at 1 kHz, digitized at 10 kHz, and analyzed offline using pClamp 10. AMPA receptor-mediated EPSC amplitude was defined as the difference between the peak response and baseline. NMDA receptor-mediated EPSC amplitude was measured as the current amplitude 50 m after the response onset. For sEPSC frequency analysis, a detection template was generated using the “Event Detection/Create Template” function, as described in (Ripoli et al., 2013). sEPSCs were then detected using the “Event Detection/Template Search” function, with a template match threshold set to 3.5. Results were manually inspected to eliminate false positives. For amplitude analysis, all waveforms detected in a single recording were averaged, and the mean amplitude was calculated. For the kinetic analysis of spontaneous excitatory postsynaptic currents (sEPSCs), only clearly isolated, non-overlapping events were analyzed. Events showing temporal overlap or occurring within bursts were excluded, as they could artificially prolong rise time measurements. Event detection and selection were performed using defined thresholds, ensuring that only clean, individual sEPSCs with stable baselines and smooth rising phases were included in the analysis.

### Statistics

Statistical comparisons and analyses were carried out with SigmaPlot software 14.0. Data samples were subjected to normal distribution assay and then expressed as mean ± standard error of the mean (SEM). For statistical comparisons we used one of the following tests: two-tailed Student’s t-test and one-way ANOVA with Bonferroni’s or Dunnet’s *post hoc* tests. The Mann–Whitney (Wilcoxon) nonparametric statistic was used when experimental data were fewer than 10 observations. The level of significance (p) was set at 0.05.

## Results

### Functional deletion of α7 nicotinic acetylcholine receptor alters NMDA receptor expression

To investigate the role of the α7-nAChR in neuronal communication, we first analyzed the expression of proteins typically associated with synaptic transmission in hippocampi of young α7-KO mice (4–6 months old) compared to wild-type (WT, C57BL/6) controls. The use of young animals is based on evidence that this age may represent a prodromal phase of neurodegenerative processes. Indeed, previous studies have shown that older α7-KO mice (12 months and above) exhibit an AD-like phenotype, including aberrant Aβ and hyperphosphorylated tau accumulation, impaired LTP at CA3–CA1 synapses and memory deficits (Cheng and Yakel, 2015).

**FIGURE 1:**
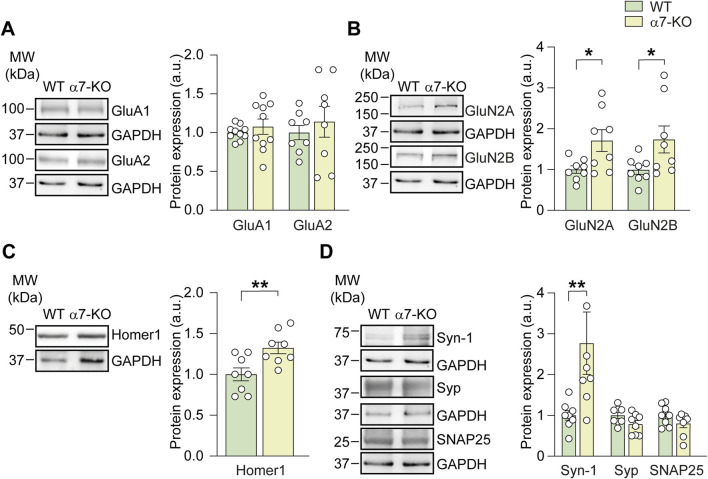
Lack of α7-nAChRs impairs the expression of pre- and post-synaptic proteins. Bar graphs and respective representative WB analysis carried out on hippocampal lysates of WT and α7-KO mice, detecting the expression of: **(A)** AMPA subunits (GluA1, n = 10/group, and GluA2, n = 8/group); **(B)** NMDA subunits (GluN2A, n = 8/group, and GluN2B, n = 8/group); **(C)** Homer1, n = 8/group; **(D)** Synapsin-1, n = 8/group, Synaptophysin, n = 8/group, and SNAP25, n = 8 WT and n = 7 α7-KO. GAPDH was used as loading control, statistical significance was assessed by Student’s t-test. *p < 0.05, **p < 0.01.

We performed Western blot analysis on hippocampal lysates from α7-KO and WT mice, focusing on AMPA and NMDA receptor subunits, the post-synaptic scaffold protein Homer-1, and presynaptic markers such as synapsin-1 and synaptophysin. AMPA receptor subunits GluA1 and GluA2 were similarly expressed in both genotypes (Figure 1A). In contrast, the NMDA receptor subunits GluN2A and GluN2B were significantly upregulated in the hippocampi of α7-KO mice compared to those of WT controls. Specifically, GluN2B expression increased from 1.00 ± 0.12 in WT to 1.75 ± 0.35 in α7-KO mice (+75%; p = 0.049; Figure 1B). Similar results were observed for GluN2A, which showed a 71% increase in α7-KO mice (from 1.00 ± 0.12 to 1.71 ± 0.28; p = 0.024; Figure 1B).

Interestingly, α7-KO mice also exhibited increased expression of the scaffold post-synaptic protein Homer1 b/c that was 33% ± 8% higher than in WT mice (p = 0.009; Figure 1C). This scaffold protein, belonging to the post-synaptic density (PSD) family, is known to i) interact with mGluR1-5 receptors, regulating their trafficking from the cytoplasm to the plasma membrane, and ii) cluster with GluN2B (Gao et al., 2013). We also found that functional deletion of α7-nAChR resulted in a significant increase in synapsin-1 expression, rising from 1.00 ± 0.16 in WT mice to 2.55 ± 0.75 in the hippocampi of α7-KO mice (p = 0.0064), whereas no differences were found for synaptophysin and SNAP25 (Figure 1D).

### Hippocampal neurons from α7-KO mice exhibit altered synaptic transmission

We then wondered whether the observed difference in synaptic protein expression in the hippocampus of α7-KO mice correlated with functional alterations in neuronal communication. To address this issue, we performed patch-clamp experiments on CA1 hippocampal pyramidal neurons in brain slices obtained from 4 to 6 months-old WT and α7-KO mice.

**FIGURE 2:**
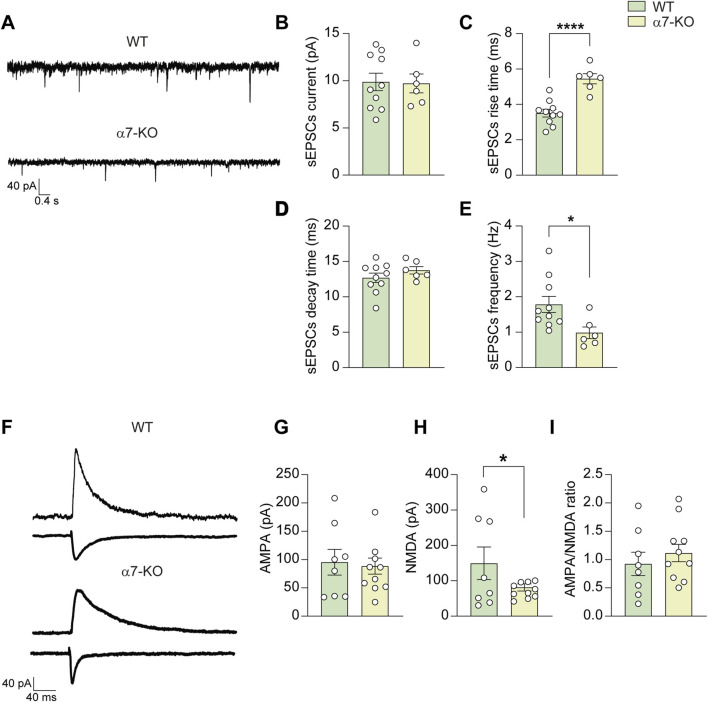
CA1 hippocampal α7-KO neurons exhibit altered synaptic transmission. **(A)** Representative traces of sEPSCs recorded in CA1 neurons (WT, top trace; α7-KO, bottom trace). **(B)** Bar graph representing the mean amplitude of sEPSCs carried out as in **(A)** (n = 10 WT; n = 6 α7-KO). **(C)** Bar graph representing the mean rise and **(D)** decay times of sEPSCs recorded as in **(A)**. **(E)** Bar graph representing the mean frequency of sEPSCs recorded as in **(A)**. **(F)** Representative AMPA and NMDA evoked currents in WT and α7-KO CA1 hippocampal neurons. Stimulation artifacts were removed for clarity. **(G–I)** Bar graphs showing the mean amplitudes of AMPA **(G)** and NMDA **(H)** currents, as well as the and AMPA/NMDA ratio **(I)** (n = 7 WT and n = 10 α7-KO for all graphs). Statistical significance was assessed by Student’s t-test. *p < 0.05, ****p < 0.0001.

Spontaneous excitatory post-synaptic currents (sEPSCs, recorded at a holding potential of −70 mV) exhibited similar amplitudes between WT and α7-KO mice (9.9 ± 1.0 vs. 9.7 ± 1.1 pA, n = 10 and 6, respectively; Figures 2A,B). However, the kinetics of sEPSCs were altered. The rise time (i.e., time to peak) was significantly longer in α7-KO neurons than in WT (5.4 ± 0.3 vs. 3.5 ± 0.2 m, respectively; p < 0.0001; Figure 2C) whereas the decay time was similar for the two genotypes (13.9 ± 0.5 and 12.7 ± 0.7 m in α7-KO and WT, respectively; p = 0.28; Figure 2D). We also observed a marked decrease in sEPSC frequency in α7-KO neurons, that was 0.97 ± 0.19 Hz vs. 1.78 ± 0.24 Hz in WT (p = 0.02, assessed by Student’s t-test; Figures 2A,E).

Evoked AMPA and NMDA currents were also examined: AMPA currents in CA1 neurons were not significantly different between WT and α7-KO mice (95.3 ± 24.3 pA and 82.2 ± 25.9 pA, respectively; Figures 2F,G) whereas NMDA currents in α7-KO mice were significantly smaller (73.2 ± 16.2 pA in α7-KO CA1 neurons vs. 163.6 ± 54.6 pA in WT, p = 0.050 Figures 2F,H). However, the AMPA/NMDA ratio differences between WT neurons (0.92 ± 0.22) and α7-KO ones (1.14 ± 0.24) did not reach statistical significance (p = 0.164; Figures 2F,I).

### Functional deletion of α7-nAChRs affects depolarization-induced glutamate release from hippocampal neurons

**FIGURE 3:**
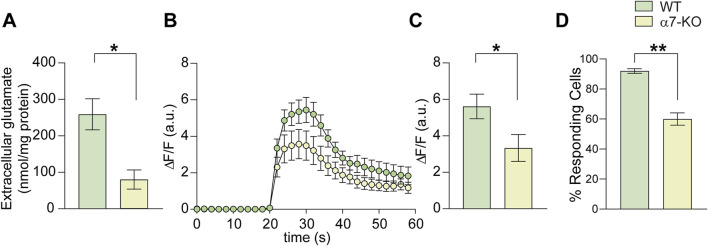
Lack of α7-nAChRs affects glutamate release and Ca^2+^ transients in hippocampal neurons. **(A)** Bar graph representing the mean amount of glutamate released extracellularly by cultured WT and α7-KO hippocampal neurons stimulated with KCl 50 mM for 30 s (n = 5 WT and n = 4 α7-KO; *p < 0.05 assessed by Mann-Whitney test). **(B)** Time course of intracellular Ca^2+^ transients elicited by 10 s KCl 50 mM stimulation in WT and α7-KO hippocampal neurons. (n = 42 for WT and n = 31 for α7-KO). **(C)** Bar graph representing the mean amplitude of intracellular Ca^2+^ transients in WT and α7-KO hippocampal neurons triggered by KCl 50 mM for 10 s and recorded for 1 min *p < 0.05 assessed by Student’s t-test. **(D)** Bar graph representing the percentage of responding WT and α7-KO neurons stimulated with KCl 50 mM for 10 s **p < 0.01 assessed by Student’s t-test.

To determine whether α7-nAChR deletion affects glutamate release from hippocampal neurons in addition to altering NMDA receptor expression, we performed HPLC experiments to quantify glutamate levels in the culture medium of WT and α7-KO primary hippocampal neurons following 30-s exposure to 50 mM KCl in Tyrode’s solution. This stimulus is indeed known to determine cell depolarization. We found that the glutamate released from α7-KO neurons was significantly smaller than that released from WT ones (80.1 vs. 259.2 nmol/mg protein; p = 0.012; n = 4 independent experiments; Figure 3A). This was associated with reduced Ca^2+^ influx upon depolarization, as evidenced by the maximum amplitude of intracellular calcium transients that was, in terms of ΔF/F, 5.6 ± 0.7 in WT vs. 3.3 ± 0.8 (p = 0.027) α7-KO neurons (Figures 3B,C). Moreover, the percentage of cells responding to depolarizing stimulus with a Ca^2+^ increase was reduced in the α7-KO cultures, decreasing from 92% ± 5% in WT neurons to 60% ± 11% in α7-KO neurons (p = 0.005, Figure 3D).

### Selective re-expression of neuronal α7-nAChRs rescues the post-synaptic alterations observed in α7-KO mice

**FIGURE 4:**
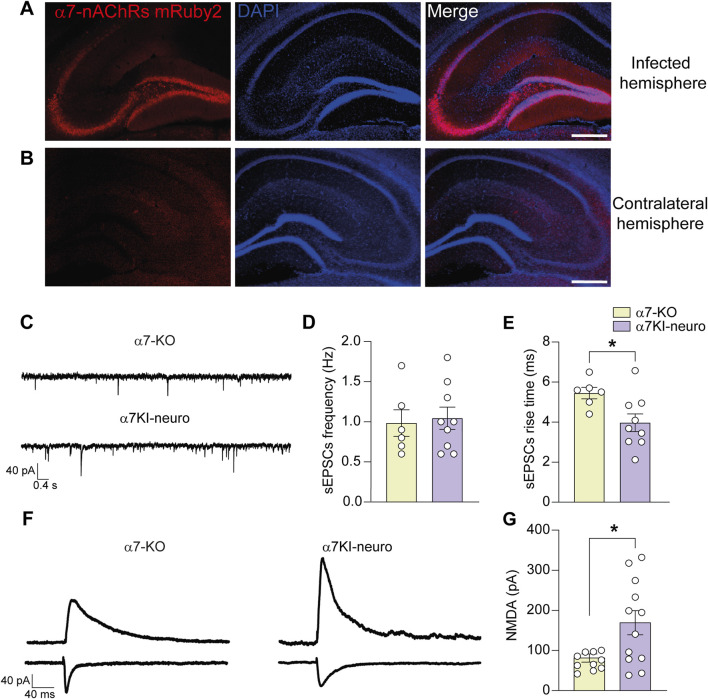
Neuronal α7-nAChR is involved in the functional post-synaptic modification of α7-KO model. **(A,B)** Representative immunofluorescence images carried out on α7-KO animals infected with the viral vector AAV2-PhP.eB-hSyn > CHRNA7-mRuby2. The mRuby2 reporter gene is detected in the infected hemisphere **(A)** while is absent in the contralateral one **(B)**; scale bar: 100 µm. **(C)** Representative traces of sEPSCs recorded in CA1 neurons from α7-KO hippocampus (top trace) and α7KI-neuro one (bottom trace). **(D)** Bar graphs representing the mean frequency of sEPSCs recorded as in **(C)** (n = 6 for α7-KO and n = 9 for α7KI-neuro). **(E)** Bar graphs representing the mean rise time of sEPSCs as shown in **(C)** (n = 6 for α7-KO and n = 9 for α7KI-neuro). **(F)** Representative NMDA evoked currents in α7-KO (left) and α7KI-neuro (right) CA1 hippocampal neurons. Stimulation artifacts were removed for clarity. **(G)** Bar graphs showing the mean NMDA currents recorded in CA1 neurons from α7-KO slices (n = 10) and α7KI-neuro ones (n = 12). Statistical significance was assessed by Student’s t-test. *p < 0.05.

Organotypic hippocampal slices obtained from WT mice and treated with AAV2-PHP.eB-hSyn > shRNA_CHRNA7-hSyn > mRuby2 (α7KO-neuro), which selectively silences neuronal α7-nAChRs, exhibited a 52% ± 15% reduction in α7-nAChR protein levels, as measured by WB (p = 0.011 vs. non-silenced WT; Supplementary Figure S1A,B). After, we selectively re-expressed α7-nAChRs in hippocampal neurons of α7-KO mice using an AAV2-PHP.eB-hSyn > CHRNA7-mRuby2 vector (α7KI-neuro model). This model allowed us to determine the specific role of neuronal α7-nAChRs in the post-synaptic modifications observed in the KO mice. Three weeks after the intra-hippocampal injection hippocampal neurons exhibit expression of mRuby2-conjugated α7-nAChRs (Figures 4A,B). No differences were found in terms of sEPSCs frequency between α7-KO and α7KI-neuro neurons (Figures 4C,D). On the contrary, the rise time of sEPSCs, that is considered a post-synaptic parameter, was restored by the specific re-expression of neuronal α7-nAChRs. Indeed, in α7KI-neuro neurons it was 4.0 ± 0.5 (p = 0.05 vs. α7-KO; Figure 4E). Also, the NMDA current amplitude, that was smaller in α7-KO neurons, was restored by the re-expression of the receptor, returning to a value of 169.2 ± 31.5 (p = 0.015 vs. α7-KO; Figures 4F,G).

## Discussion

Alpha7-nicotinic acetylcholine receptor (α7-nAChR) is a homopentameric receptor for acetylcholine highly expressed in brain areas fundamental to memory and cognition, such as the amygdala and the hippocampus (Letsinger et al., 2022), mainly on pyramidal neurons. From a functional point of view, activation of α7-nAChRs induces cell depolarization and modulates both cholinergic and glutamatergic transmission by influencing pre‐ and post-synaptic mechanisms, thereby affecting neurotransmitter release and post-synaptic currents. The high Ca^2+^ permeability of α7-nAChR strongly impacts on gene transcription, especially at neuronal level. Given the importance of α7 receptor activity, its dysfunction may lead to several illnesses. For example, reduced α7-nAChR function is associated with schizophrenia, as the expression of α7-nAChR is decreased in hippocampus and other brain regions of schizophrenic patients (Freund et al., 2016). Moreover, stimulation of nicotinic acetylcholine α7 receptors with specific agonists rescue schizophrenia-like cognitive impairments in rodents (Potasiewicz et al., 2017). These receptors play also a crucial role in hippocampal synaptic plasticity (e.g., LTP at CA3-CA1 synapse) by regulating the release of glutamate. Aβ42 has been reported to act as α7-nAChR agonist thus regulating synaptic plasticity in healthy brain (Hascup and Hascup, 2016). Deletion of α7-nAChR function has been proposed to trigger aberrant Aβ accumulation and the onset of an AD-like pathology (Tropea et al., 2021). Indeed, α7-KO mice older than 12 months exhibited increased Aβ levels, associated to tau hyperphosphorylation, neuronal loss and astrogliosis, all representing molecular hallmarks of the disease (Tropea et al., 2021).

Based on these findings, we investigated whether α7-KO mice, at an age of 4–6 months likely representing a prodromal phase of the disease, already show early signs of glutamatergic dysfunction at hippocampal level.

Even if at this age no accumulation of Aβ and pTau was observed, hippocampal neurons of α7-KO mice exhibited altered expression of pre- and post-synaptic proteins, including synapsin-1 and the NMDA receptor subunits GluN2A and GluN2B, along with the post-synaptic scaffold protein Homer-1, that were increased. These molecular changes were associated with significant alterations of the electrophysiological properties of the CA1 pyramidal neurons, including the frequency of spontaneous EPSCs, the time to peak of these currents, and the amplitude of evoked glutamate-mediated NMDA currents. Conversely, no significant changes were observed in AMPA receptor-mediated transmission, at both molecular and functional levels. The prodromic stage of the AD pathology is also characterized by alteration of calcium dynamics, which contributes to disrupted function of glutamatergic transmission. Accordingly, we found that α7-KO neurons also exhibited altered depolarization-induced Ca^2+^-transients and reduced glutamate release.

In neurons, ionotropic glutamate receptors and nAChRs are actually colocalized and functionally interdependent (Stone et al., 2021). Specifically, the formation of nAChR and NMDA receptor complexes promoting glutamate release have been reported by Li and colleagues (Li et al., 2012). This interaction is further supported by the regulation of protein trafficking including subunits of glutamate receptors carried out by α7-nAChRs (Puddifoot et al., 2015). The activation of α7-nAChRs can also modify the subunit composition as well as cellular distribution of both NMDA and non-NMDA receptors, leading to altered neuronal excitability (Stone et al., 2021). The discrepancy between the increased expression of NMDA subunits and the reduced amplitude of NMDA currents might be due to not fully functional NMDARs, thus not allowing proper cation permeability, as suggested by previous literature reports demonstrating a functional interaction between the α7‐nAChRs and NMDAR expression and function (Abraham et al., 2025). Alternatively, NMDARs might be retained in the cytosol, thus precluding their functional insertion in the plasma membrane. At the same time we also can explain the reduced frequency of sEPSCs with the increased expression of synapsin-1 and the decreased depolarization-induced Ca^2+^ entry in neurons. Indeed, synapsin is known to bind synaptic vesicles to actin filaments in the cytoskeleton thus increasing the “reserve” pool. We hypothesize that: i) synapsin overexpression increases the reserve pool and reduces the readily releasable pool; ii) the reduction in intracellular Ca^2+^ transient amplitude following neuronal depolarization may also limit CaMKII-mediated synapsin phosphorylation, leading to reduced number of SVs at the presynaptic terminal, thus affecting spontaneous vesicular release. In 2013, Lin and colleagues studied the age-dependent modification of NMDA receptor subunits observed at cortical level by α7-KO mice. In particular, they reported that the major differences respect to WT mice were observed in the first 2 months of age (8–56 days postnatal). In line with Lin et al.'s findings, we found that at 5 months of age NR2B expression in the cortex of α7-KO mice is slightly, though not significantly, reduced (Supplementary Figure S2). In contrast, NR2B expression in the hippocampus of the same animals is increased.

Given that α7-nAChRs are expressed in both neurons and astrocytes (Fontana et al., 2023), we sought to dissect the specific contribution of neuronal α7 receptors to the observed alterations in glutamatergic transmission by focusing on electrophysiological readouts. By a specific adeno-associated viral (AAV) vector-based silencing system, we found that in WT mice, neuronal α7-nAChR accounted for half of the total receptors, supporting the rationale underlying our study of a key contribution of neuronal α7‐nAChRs in the alteration of glutamatergic synaptic transmission. We thus employed an AAV vector engineered to selectively re-express α7-nAChRs in hippocampal neurons of α7-KO mice. This was achieved by driving CHRNA7 expression under the control of the neuron-specific human synapsin promoter (α7KI-neuro).

Following hippocampal AAV injection, we found that the re-expression of neuronal α7-nAChRs was sufficient to rescue post-synaptic alterations, but not presynaptic ones. Specifically, 3 weeks post-AAV injection, CA1 pyramidal neurons of α7KI-neuro mice displayed normalized NMDA receptor-mediated current amplitude and restored rise time of spontaneous EPSCs. However, the decreased frequency of sEPSCs remained unchanged, indicating that the presynaptic deficits were not rescued by neuronal α7-nAChR expression alone.

In conclusion, our findings suggest that the functional loss of α7-nAChRs disrupts glutamatergic transmission through distinct mechanisms acting at pre- and post-synaptic levels, with neuronal receptors playing a pivotal role in maintaining post-synaptic integrity. This highlights the complexity of α7-nAChR signaling in the hippocampus and supports the idea that early synaptic dysfunction in AD may arise from the disruption of cholinergic-glutamatergic cross-talk well before overt neurodegeneration occurs. Disentangling the cell-type-specific contributions of α7-nAChRs could therefore open new routes for targeting prodromal synaptic failure in AD and refining therapeutic strategies aimed at preserving circuit function in the early stages of the disease.

## Data Availability

The raw data supporting the conclusions of this article will be made available by the authors, without undue reservation.
