# Supplementary material for: Functional deletion of α7 nicotinic acetylcholine receptor impairs Ca2+-dependent glutamatergic synaptic transmission by affecting both presynaptic and postsynaptic protein expression and function
Source: Front Physiol. 2025 Aug 8;16:1662171. doi: 10.3389/fphys.2025.1662171 (PMC12370769; doi:10.3389/fphys.2025.1662171)
Supplement: Supplementary file 1 [file Supplementaryfile1.docx]

**Functional deletion of α7 nicotinic acetylcholine receptor impairs Ca^2+^-dependent glutamatergic synaptic transmission by affecting both presynaptic and postsynaptic protein expression and function**

Beatrice Cannata^1,2§^, Laura Sposito^1§^, Martina Albini^1^, Giuseppe Aceto^1,2^, Giulia Puliatti^1^, Giacomo Lazzarino^3,4^, Cristian Ripoli^1,2^, Maria Rosaria Tropea^5^, Daniela Puzzo^5,6^, Roberto Piacentini^1,2^* and Claudio Grassi^1,2^

1. Department of Neuroscience, Università Cattolica del Sacro Cuore, 00168 Rome, Italy
2. Fondazione Policlinico Universitario A. Gemelli IRCCS, 00168 Rome, Italy
3. Departmental Faculty of Medicine, UniCamillus - Saint Camillus International University of Health Sciences, 00131, Rome, Italy
4. IRCCS San Camillo Hospital, 30126 Venice, Italy
5. Department of Biomedical and Biotechnological Sciences, University of Catania, 95123 Catania, Italy
6. Oasi Research Institute-IRCCS, 94018 Troina, Italy


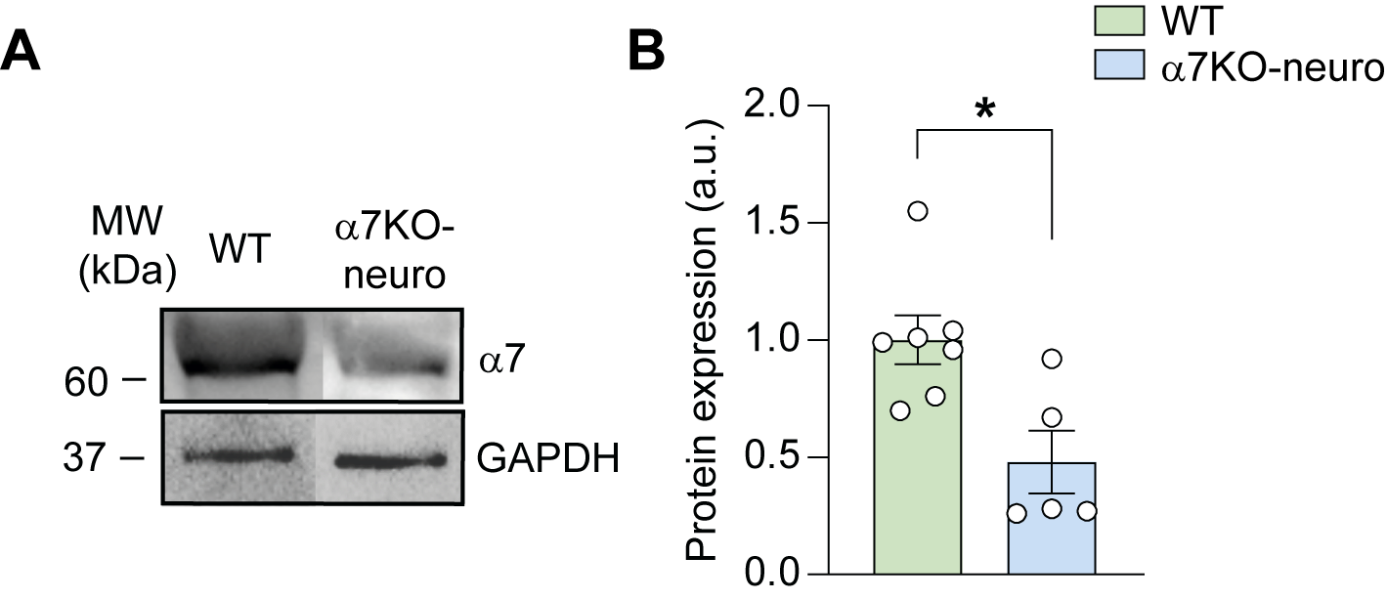


**Figure S1. *Ex vivo* silencing of neuronal α7-nAChR halved the quantity of the receptor in WT organotypic hippocampal slices**. **(A)** Representative WB analysis carried out on WT, α7KO-neuro organotypic hippocampal slices, detecting the expression of α7-nAChR (n=7 and 5, respectively). **(B)** Bar graph quantifying the expression of the α7-nAChR as in panel (A).


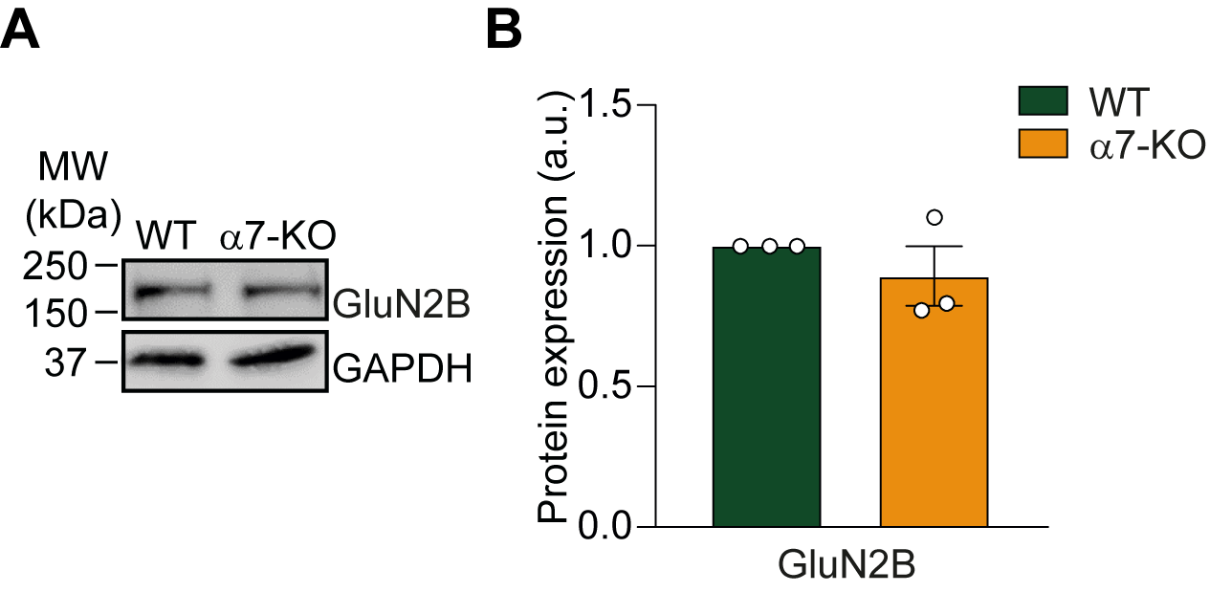


**Figure S2. GluN2B expression in neocortex of 4-month-old α7-KO mice.** **(A)** Representative WB analysis carried out on cortical lysates of WT and α7-KO mice, detecting the expression of GluN2B. **(B)** Bar graphs quantifying WB experiments as in panel A (n=3/group).
